# Inferring diet, disease and antibiotic resistance from ancient human oral microbiomes

**DOI:** 10.1099/mgen.0.001251

**Published:** 2024-05-13

**Authors:** Gwyn Dahlquist-Axe, Francesca J. Standeven, Camilla F. Speller, Andrew Tedder, Conor J. Meehan

**Affiliations:** 1 School of Chemistry and Biosciences, University of Bradford, Bradford, UK; 2 Department of Anthropology, University of British Columbia, Vancouver, Canada; 3 Department of Biosciences, Nottingham Trent University, Nottingham, UK

**Keywords:** ancient DNA, antibiotic resistance, evolution

## Abstract

The interaction between a host and its microbiome is an area of intense study. For the human host, it is known that the various body-site-associated microbiomes impact heavily on health and disease states. For instance, the oral microbiome is a source of various pathogens and potential antibiotic resistance gene pools. The effect of historical changes to the human host and environment to the associated microbiome, however, has been less well explored. In this review, we characterize several historical and prehistoric events which are considered to have impacted the oral environment and therefore the bacterial communities residing within it. The link between evolutionary changes to the oral microbiota and the significant societal and behavioural changes occurring during the pre-Neolithic, Agricultural Revolution, Industrial Revolution and Antibiotic Era is outlined. While previous studies suggest the functional profile of these communities may have shifted over the centuries, there is currently a gap in knowledge that needs to be filled. Biomolecular archaeological evidence of innate antimicrobial resistance within the oral microbiome shows an increase in the abundance of antimicrobial resistance genes since the advent and widespread use of antibiotics in the modern era. Nevertheless, a lack of research into the prevalence and evolution of antimicrobial resistance within the oral microbiome throughout history hinders our ability to combat antimicrobial resistance in the modern era.

## Introduction

Over 1.5 billion years, coevolution between micro-organisms and their hosts has resulted in reciprocal adaptation and functional integration, as shown in our own interaction with the majority of micro-organisms that populate our body surfaces [1]. In this sense, for as long as humans and their ancestors have existed, we have been living and evolving alongside the microbiota that live in and around our bodies, simultaneously shaping one another’s survival strategies. Therefore, studying the ancient human microbiome and its constituents can aid our understanding of the evolutionary networks belonging to both humans and micro-organisms.

In host-associated microbiomes, microbiota perform critical tasks that determine and contribute to the host’s physiology, establishing a unique biological interaction known as symbiosis [2,3]. Host–microbiome symbiosis is an important benefactor for general human health. It regulates the cardiovascular system, aids host defence functions, harbours inflammatory properties, has metabolic potential and antioxidant activity, supports a healthy digestive tract, and donates resistance to pathogen colonization [1]. However, imbalance in the structure of the microbiome can lead to detrimental functional changes that harm the host (referred to as dysbiosis), which can be linked to various diseases and infection [4].

The human microbiome consists of numerous communities of bacteria, archaea, viruses, fungi, protists and other micro-organisms. Microbial communities are located in various areas of the human body including the nose [5], vagina [6], oral cavity [7], skin [8] and gastrointestinal tract – the last being the most widely researched area of the human microbiome [9] and which maintains the largest concentration and complexity of micro-organisms in the body [10]. Each microbial community is distinct in its compositions and functions within the human body [11]. The communities of bacteria across the human body engage in a variety of interactions with their environment allowing them to efficiently respond to changes. The adaptive potential of human microbiome communities is key to their ability to maintain dominance over other species or communities also vying for territory in fluctuating environments [12].

One environmental stress that residents of the human microbiome must often deal with is the influx of antibiotics for treating pathogenic infections. Antibiotic misuse, overuse and underuse tend to increase antibiotic load in the environment, leading to the evolution of antibiotic resistance in microbial communities [13]. It was during the 1940s when hospitals began to detect early warning signs of an increase in penicillin- and tetracycline-resistant micro-organisms [14]. Now, the World Health Organisation declares antimicrobial resistance (AMR) a global health crisis [15], which has an annual mortality prediction of 10 million lives by 2050 [16].

The spread of AMR demonstrates a vast and intricate network of linked transmission pathways that connect humans to a variety of environments such as hospitals, sewage, farms and the wider ecosystem including wildlife [17]. Antibiotic resistance genes (ARGs) are genes belonging to bacteria that allow them to confer resistance to certain antibiotics. Many ARGs are present on transposons, integrons or plasmids, which can be mobilized and passed on to bacteria of the same or different species [18]. Resistance can result from mutations or the acquisition of resistance-conducting genes via horizontal gene transfer, with the latter contributing significantly to AMR [19]. Bacteria can develop resistance in a variety of ways. Acquired resistance is the transfer of genes or acquisition of mutations that cause bacteria to become resistant to a specific antibiotic [20]. Intrinsic resistance is the ability to resist drugs due to innate structural or functional characteristics [21]. Silent resistance genes are rarely expressed in wild-type bacteria and proto-resistance genes are not capable of generating resistance; however, mutations can change or increase their spectrum of action [22].

**Fig. 1.**
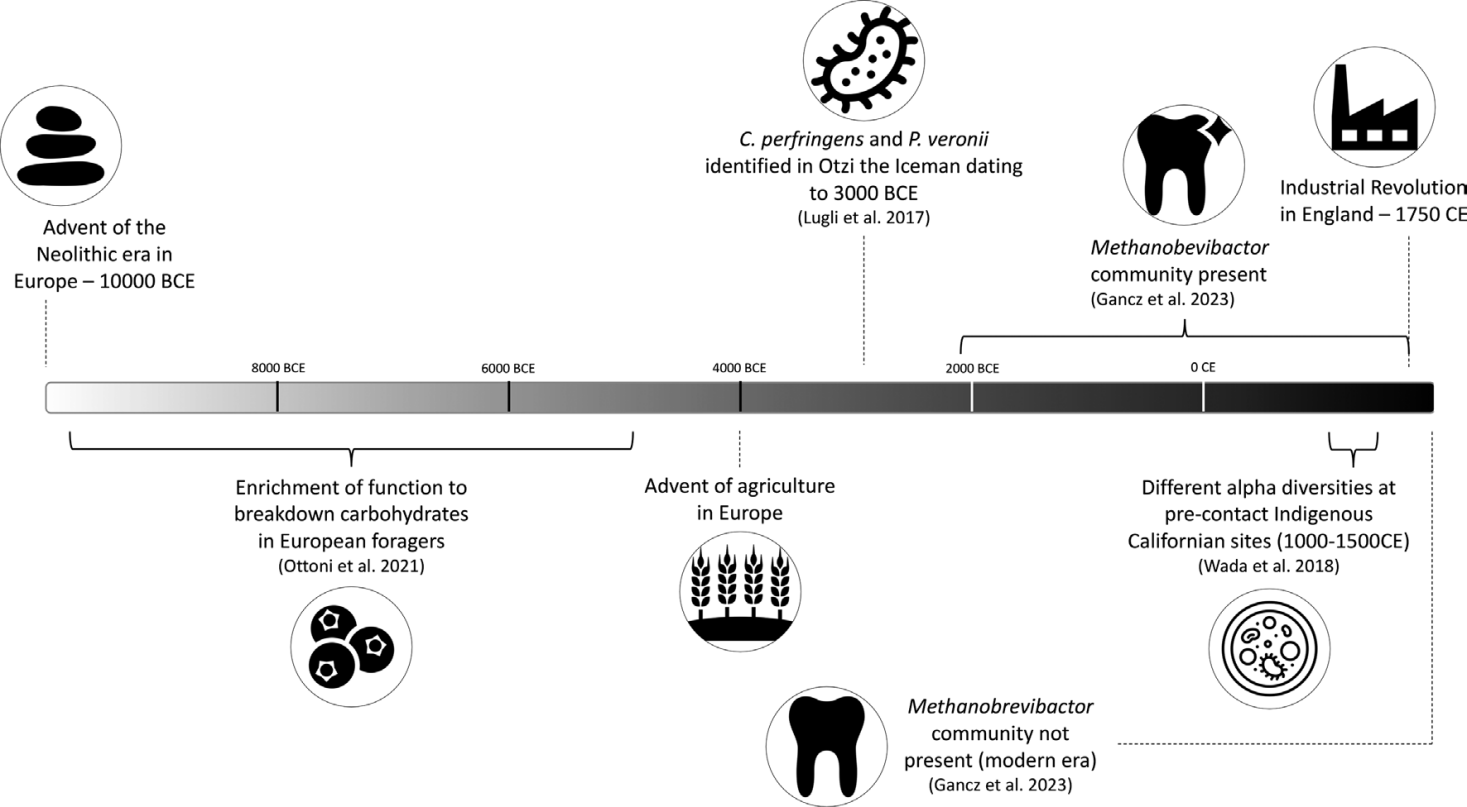
Timeline of notable events in human history pertaining to diet and the evolution of the oral microbiome.

Through extensive study of the bacterial communities that constitute various aspects of the human microbiome, links have been established between specific internal and external factors and changes to the composition or abundance of species in the human microbiome as well as their function [23,30]. Knowing that a change in host lifestyle can result in a change in the microbiome, it is important to understand what changes will occur given the introduction of new dietary resources, new environmental exposures or access to new medicines, such as those that occurred during the pre-Neolithic Era, the Agricultural Revolution, the Industrial Revolution and the Antibiotic Era (Figs1^3^). This review aims to address our current understanding of how oral bacterial communities responded to the changes that occurred across thousands of years of human societal evolution and how we can use this knowledge of the past to inform the present and future of bacterial research.

**Fig. 2.**
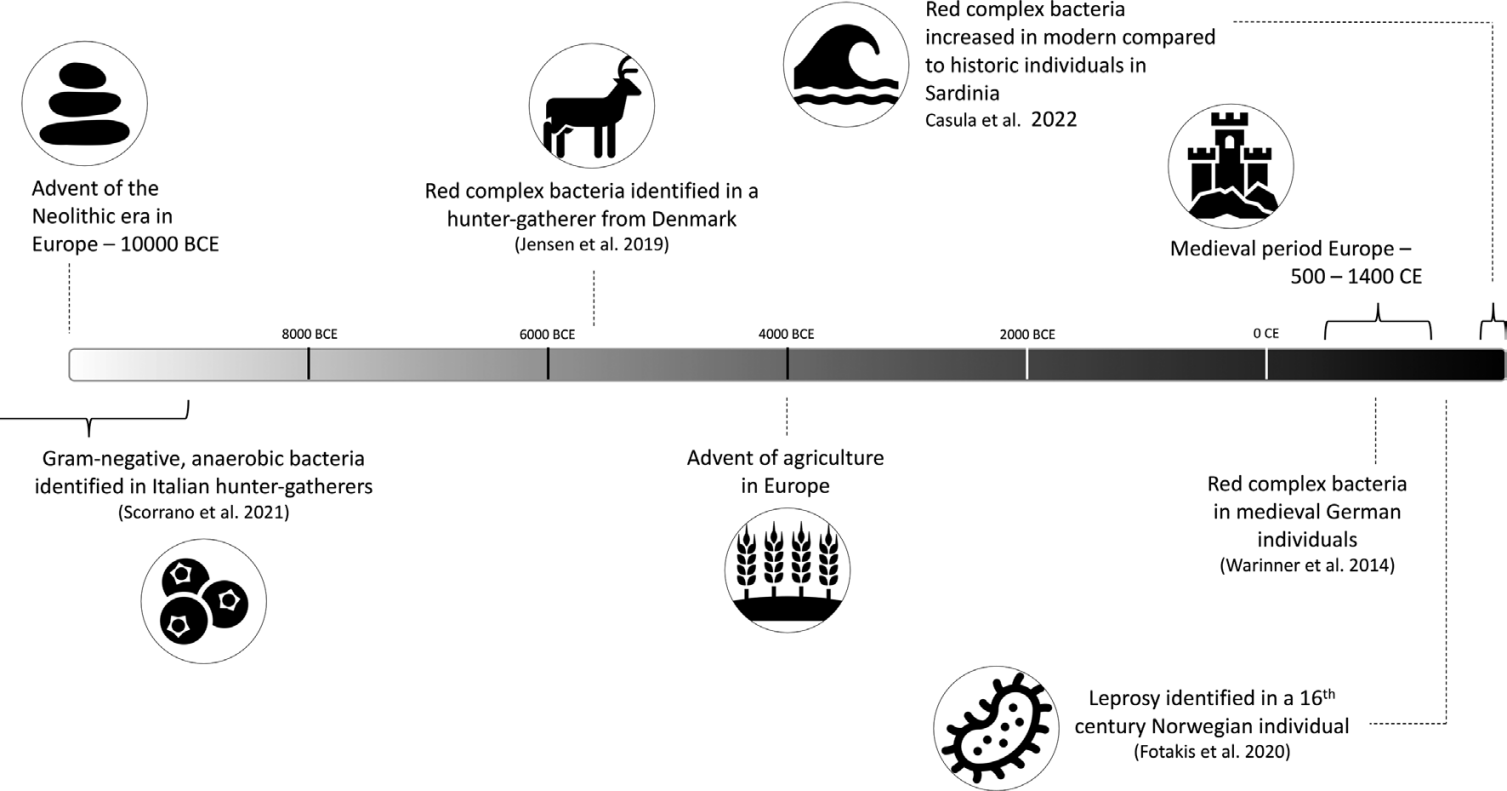
Timeline of notable events in human history pertaining to disease and the oral microbiome.

**Fig. 3.**
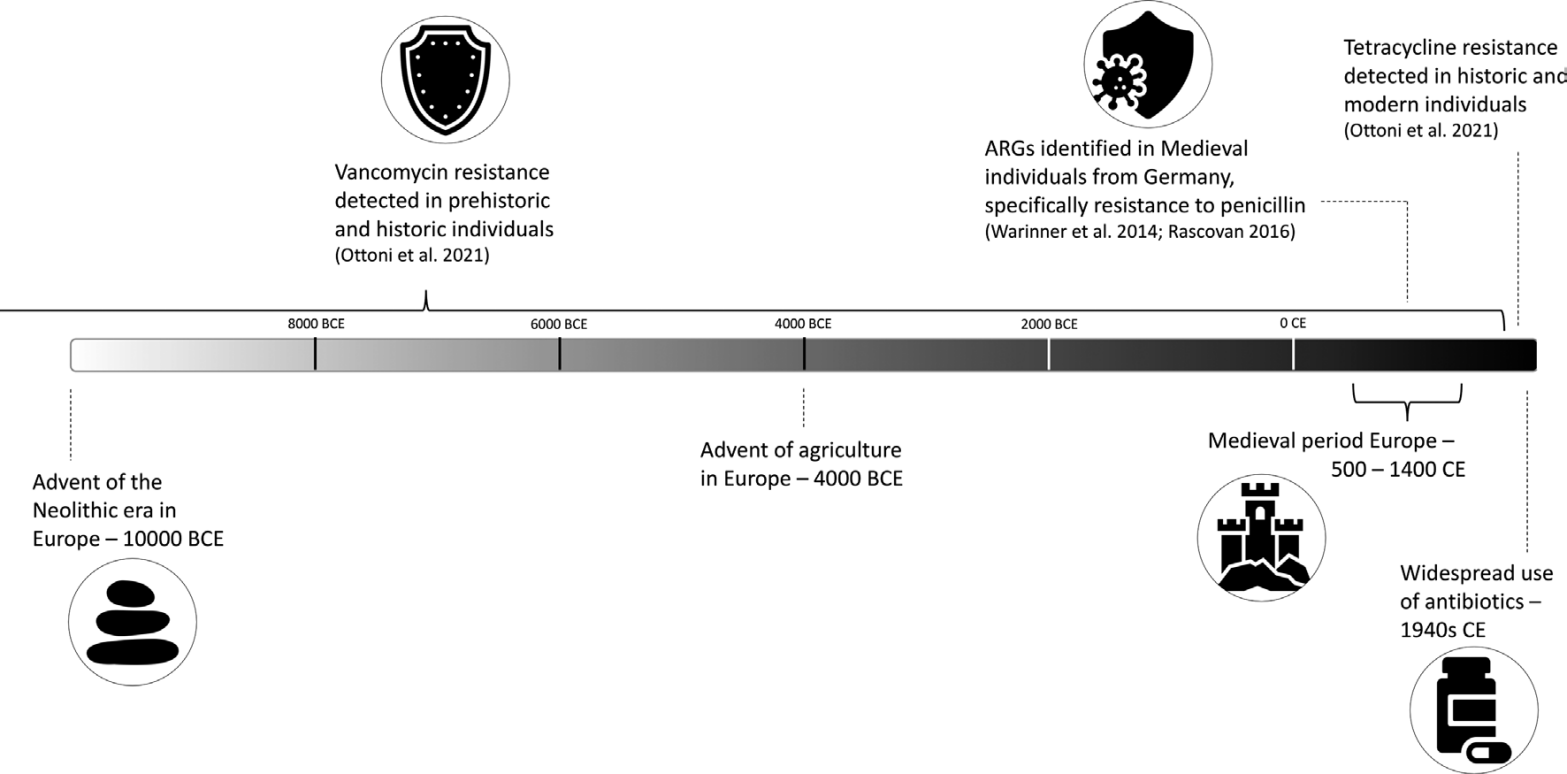
Timeline of notable events in human history pertaining to the evolution of antimicrobial resistance in the oral microbiome.

## The oral microbiome

Human oral microbiomes encompass an array of microbial species whose functions and interactions with the host can impact human health. The oral cavity is known to accommodate the second most diverse microbial community in the body, containing over 700 bacterial species that populate the hard and soft tissues of the mouth [1]. Just 1 ml of saliva holds around 10^8^ microbial cells with an approximate range of 100–200 distinct bacterial organisms in a typically healthy microbiome [31]. Core bacterial genera present in ≥66 % of healthy human oral cavities include *Streptococcus, Granulicatella, Neisseria* and *Haemophilus* [32]. Healthy oral microbiomes are dominated by *Firmicutes*, *Proteobacteria*, *Actinobacteria*, *Bacteroidetes*, *Fusobacteria* and *Spirochaetes* [33], whereas *Porphyromonas gingivalis, Tannerella forsythia* and *Treponema denticola* (red complex) [33,34] and *Streptococcus mutans*, lactobacilli and *Actinomycetes* species are linked to disease [35].

The inter-bacteria abundance dynamics within the oral cavity are complex and it is believed that community composition is strongly linked to perturbations, often leading to dysbiosis [36]. As different bacterial species thrive in different conditions, some are expected to dominate whilst others diminish depending on nutrient availability [37]. It is dietary sugars that are often blamed for dental disease in ancient and modern humans, including caries and periodontal disease [38,39]. Carbohydrates, specifically sugars, supply many species with the necessary nutrients to colonize and thrive, especially pathogenic bacteria [37], such as *S. mutans, Veillonella dispar* and *Actinomyces gerencseriae,* which are linked to biofilm formation and caries [40]. Bacteria associated with good oral health, such as *Streptococcus mitis*, are more successful in low-sugar environments [41, 42]. These species working within diverse communities can colonize surfaces in the mouth and can hinder pathogens from binding and multiplying [148].

Selective forces have created habitat-specific subgroups of bacterial species whose genomes display adaptation to niches within the oral cavity [43]. Due to density and genetic diversity within bacterial communities, a large variety of aggressive behaviours have evolved among bacteria including antibiotic and toxin production, and mechanical weapons [44], such as the type VI secretion system that acts as a ‘nano-crossbow’ to propel a spike-tipped tube to deliver protein effectors into target cells [45]. Conversely, bacteria can work together in communities against external stimuli, which can benefit the community even when some members are harmed [46]. Oral bacterial communities may recruit new species if a close relative of that species is not already present [47]. This indicates competition between closely related species and community-level selection for diversity [47], suggesting that multiple species from the same genera are unlikely to be found within the same oral community. This gives insight into which oral bacterial communities respond to the loss or addition of new species and the functions they perform. For a bacterial community to thrive, a variety of functions must be performed by community members, and so environmental changes that drive species loss or gain require a response from the whole community [48].

The composition and functions of a bacterial community influence human health. Some communities perform functions that cannot be carried out by human cells, such as the production of certain vitamins [49], demonstrating symbiosis between humans and microbes. However, oral and general health issues can occur if the host’s normal oral microbiota is disturbed [1]. Small numbers of opportunistic pathogens can coexist alongside commensal bacterial without affecting the health of the host; however, beneficial bacterial populations can become overwhelmed when harmful bacteria proliferate, leading to functional changes and dysbiosis [50]. This inbalance is linked to a range of oral [7] and systemic diseases [51], as well as mental health [52], and can be influenced by lifestyle factors such as diet or smoking [53].

Unique microenvironments within the the oral cavity vary in bacterial composition [54], including the mouth, teeth, tongue, cheeks and gums, which offer different physical and chemical properties to disparate microbial communities [55]. Salivary flow [56] and shedding and non-shedding surfaces [57] are selective forces that help certain bacteria to colonize specific environments, resulting in localized bacterial communities. Dental plaque is a biofilm that forms on teeth and goes through several stages of maturity before mineralization (calcified plaque or dental calculus). *Actinomyces* is dominant during early biofilm formation [35,58] and plaque that is rich in *Streptococcus,* another early colonizer, is associated with good oral health [59]. Late plaque maturation incorporates higher abundances of pathogenic bacteria [60,61]. The preservation of microbial ancient DNA (aDNA) belonging to these species in dental calculus allows for the unique examination of human oral microbiome evolution. Dental calculus has proven to be a rich source of ancient biomolecules, preserving ancient oral DNA for tens of thousands of years and therefore allowing for the study of oral microbiomes from ancient individuals, including Neanderthals [62].

It is noteworthy that there are microbial differences between calculus and plaque regarding species preservation bias and the maturity of biofilms [63]. It is also important to briefly highlight the research bias in modern human oral microbiomes due to a large focus on industrialized, high-income countries rather than the Indigenous and racialized communities [64] that usually represent the highest disease burden [65].

## The function of ARGs in the modern oral microbiome

Since ancient dental calculus has the great ability to preserve bacterial genes, it allows us to investigate the ancient functions of micro-organisms. From studies of modern human oral microbiomes we have been able to determine many functions performed by oral bacteria that can be both beneficial and detrimental to the human host. Beneficial functions vary in expression including the production of cancer-prohibiting substrates [66,67], metabolism of nitrates for the production of beneficial nitric oxide [68] and pH regulation to protect against acidity [69]. Conversely, oral bacteria can perform functions that have a negative impact on the human host, such as causing inflammation that can have health impacts across the host’s whole body [70] and pathogens with ARGs that enable them to persist despite medical intervention [71,72].

It is acknowledged that some areas of AMR and oral microbiome research are understudied and, and unlike the gut, the oral microbiome is thought to be an unexplored source of ARGs [73]. Since drug resistance in oral bacteria is a growing concern in dentistry [74], the study of resistance genes in the context of oral microbiomes is medically relevant and merits exploration. ARGs are plentiful in the modern human oral cavity, increasing the likelihood of resistant bacterial infections [71]. One of the most common ARGs found in the oral cavity today are those that confer tetracycline resistance [75,80]. A high proportion of resistance genes probably originate from streptococci, which are abundant in the oral cavity [81]. ARGs among oral bacteria not only jeopardize antibiotic therapy for oral infections, but they also prevent the treatment of other diseases that may occur in the body due to bacterial translocation [71]. For instance, oral commensal streptococci serve as a reservoir of resistance genes that are utilized by the pathogen *Streptococcus pneumoniae*, potentially obstructing treatment of serious systemic infections [82]. Based on a study that found an overlap in the oral cavity and stool samples in nearly half (45 %) of individuals in the Human Microbiome Project [83], oral bacterial translocation to the rest of the body, particularly to the gut, is thought to be a common occurrence [84]. It is worth noting that there is still ongoing discussion about whether shared taxa between the oral cavity and the gut is normal in healthy humans. According to some investigations, oral strains from diverse taxa are present in the gut microbiomes of many healthy individuals [85]. On the other hand, a rise in oral bacteria in the gut is thought to indicate poor dental health, such as plaque formation [86]. Furthermore, another investigation revealed no indication of shared gut and oral bacteria in healthy people, implying that if similar taxa are discovered, they should be clinically investigated for signs of disease [87].

The presence and translocation of ARGs in human oral microbiomes is undoubtedly due to anthropogenic influence. Of course, we do not know the extent to which ARGs naturally occur in oral microbiomes, which is why we need clean models (untouched by human-made antibiotics) such as ancient microbiomes unaffected by widespread AMR to investigate questions like this. If we study the presence of ARGs in clean models, such as ancient dental calculus, or even translocation using ancient coprolites from the same site, we can begin to understand their natural diversity and spread to predict the behaviours and responses of these bacteria conferring resistance after the long-term application of new, alternative medicines, such as the use of bacteriophages [88], vaccines [89] or commensal therapy [90].

The ways in which we are able to study the human oral microbiome in general and ARGs specifically in modern contexts relies on knowledge of prokaryote genetics and technological advancements that enable the identification and analysis of bacterial DNA. In ancient contexts, however, the study of oral bacteria genetics and evolution becomes more complicated.

## Obstacles to investigating ancient oral microbiomes

Early studies attempting to retrieve ancient oral microbial DNA from archaeological dental calculus using 16S rRNA amplicon-based sequencing were hindered by the inherent biases of 16S sequencing on degraded, short-read aDNA [91]. Metagenomic characterizations of microbiomes have developed significantly in the past years with a move from 16S sequencing to whole genome shotgun sequencing (WGS). These advancements in DNA sequencing technologies allowed for investigations of the aDNA preserved in dental calculus to increase in depth, from phylum-level characterizations of ancient oral microbiomes [92] to the discovery of genes with previously unknown metabolic functions [93]. For example, analysis of the more than 5000-year-old mummified gut tissues of Otzi the Iceman using WGS successfully reconstructed the gut microbiome to the species level with additional analysis of functional profiles and virulence factors of genes in several pathogenic species [94]. An earlier study of Otzi using 16S sequencing was able to determine bacterial phylogeny within the ancient gut environment but not bacterial function [95]. This example shows how the progression of technology has impacted the specificity of data now available in the study of archaeogenomics and specifically ancient human microbiomes. While fragmentation and depurination of aDNA still pose obstacles to the study of ancient oral microbiomes, advancements have been made that reduce such barriers.

A significant factor hindering the success of ancient microbial DNA sequencing and subsequent analysis is contamination. Contamination of ancient oral microbial DNA preserved in dental calculus can be introduced by modern human intervention during excavation and laboratory analysis [96] as well as from soil and decomposition processes in the burial environment [97]. However, even with the use of protective gear during excavation and sampling as well as decontamination protocols in labs, contaminant DNA can be present in sequenced aDNA and during subsequent analyses [98]. The inclusion of negative controls during DNA extraction and library building are integral to identifying lab-based contaminants [99,100]. The ability to identify contamination in aDNA sequences is vital to successfully study ancient microbiomes. Contamination is known to artificially inflate alpha diversity in ancient dental calculus samples, highlighting the importance of thorough decontamination [101]. There are a range of computational tools designed to identify [102,103] or remove contamination from both modern and ancient sources (Duitama et al. 2023; 104) [56]. However, the ability to remove both modern and ancient contaminant DNA during computational analyses requires a control sample from both modern contexts (e.g. lab) and ancient sources (e.g. burial environment). When ancient oral metagenome research is conducted on archaeological material that was excavated or collected without the intention of analysing aDNA the control samples for contaminants in the burial environment can be missing. Resulting microbial profiles from studies that do not include an environmental control sample can contain contaminants from ancient soil environments that cannot be identified via damage authentication tools [97].

## The oral microbiome through the ages

Advancements in analytical technology have allowed for the solution of many problems faced by researchers of ancient oral microbiomes and therefore there has been an influx in meaningful research on this topic in the last decade. A prominent theme in ancient oral microbiome research is the study of interactions between human behaviour and the health state of the oral cavity, such as changes in human diet and oral disease across human history. Additionally, technological advancements have allowed for detailed research into the individual genes involved in the complex community dynamics of ancient oral metagenomes, such as AMR. The following sections focus on these themes and the current state of knowledge.

### Diet

Some correlations have been found between diet and the modern oral microbiome, specifically linking vitamin B, C and E intake to increased presence of bacterial species in the fusobacteria class [105]. Links have also been found between carbohydrate consumption and increased abundance of cariogenic bacteria such as *S. mutans* [106] as well as decreased alpha diversity [107,108]. While these studies are conducted on modern, living people, we can use the knowledge of these links to extrapolate potential similar links in ancient humans. Understanding how oral microbiomes responded to changing diets is an important step in understanding the evolution of oral bacterial communities and their functions in the human oral cavity.

Hunting and gathering is the oldest subsistence strategy in human history, and pre-dates the emergence of *Homo sapiens* [109]. Hunter-gatherers have been described as individuals who do not intentionally modify the gene pool of exploited resources, as opposed to those who rely primarily on an agricultural or pastoralist subsistence [110]. The diets of hunter-gatherers can be inferred by dental morphology [111], stable isotope analysis [112,113], and analysis of faunal remains [114], and it is largely agreed that hunter-gatherers relied on a diverse range of foodstuffs which varied depending on geographical location and seasonal period [115]. Comparisons of ancient humans, Neanderthals and non-human primates showed that the composition of the core oral microbiome has remained stable for potentially millions of years, but the functions performed by oral microbiota have changed over time as they adapted to changing host diet [116]. An example of potential dietary impacts to the oral microbiome of hunter-gatherers can be seen in pre-colonization indigenous North Americans. Analysis of dental calculus presented an interesting case of site-level diversity, possibly linked to social status and access to diverse dietary resources. Individuals from one site displayed higher abundances of *Enterococcus faecalis* and *Enterococcus faecium* while individuals from another site displayed higher abundances of *Pseudopropionibacterium porpionicum*, as well as variable alpha diversities [117].

Different populations across the globe transitioned from hunter-gatherer to an agricultural subsistence strategy at varying times, if at all (Fig. 1). Agricultural practices are characterized by the purposeful cultivation of crops and animal husbandry [118], and were marked by an overall decline in oral health [119]. Farming and hunter-gathering coexisted in many circumstances, opening up the potential to study the impacts of agricultural economies on oral microbiomes [120]. Investigation of the impact of the transition to agriculture in the Balkans and Italian Peninsula on the human oral microbiome revealed a functional shift in bacterial genes [121]. There was no significant difference in the functional profiles of foragers and farmers from this region, but foragers had an enrichment of a pathway associated with the breakdown of carbohydrates, indicating a highly plant-based diet [121].

Migration regions and the relatively slow uptake of agriculture may be reasons for the lack of differentiation between foragers and farmers in southern Europe and therefore inclusion of modern studies where such variables can be controlled for can clarify these issues. Three pairs of modern communities practising hunter-gatherer and traditional farming subsistence strategies living in close proximity in the Philippines provide such an opportunity. The hunter-gatherer population had additional gene functions linked to vitamin B production, which could be driven by less vitamin B5 in the hunter-gatherer diet [122]. The inclusion of modern individuals with typical western diets shows oral microbiomes with gene functions linked to pH regulation that buffers biofilms against acidification [122], potentially caused by the increase in sugar consumption in the western diet and the resulting changes in the oral environment.

Following the adoption of agricultural subsistence strategies, the next cultural adaption that significantly impacted diet was the Industrial Revolution. In Britain, the technological advancements that were introduced during the Industrial Revolution allowed for new and diverse foods to be mass produced and widely distributed, as well as bring in new ingredients through increased trade [123] (Fig. 1). It is firmly established by stable isotope studies [124], written records such as cookbooks [125,126] and archaeological investigations of human remains [127] that the food resources available and the average individual’s diet during the Industrial Revolution was remarkably different than earlier and later diets. In particular, dietary sugars and processed food consumption increased [128]. The effects of consuming dietary sugars on modern microbiomes are thought to include increased abundance of pathogenic bacteria and gene function specific to the uptake of sugars [129], whereas the effects of processed foods on the human oral microbiome are currently less well known.

In a widespread investigation of ancient British individuals dating from approximately 1000–1853 CE, a distinct *Methanobrevibacter*-associated community was discovered as a stable component of the ancient human oral microbiome, but thus far is missing from modern human oral microbiomes [130]. A *Streptococcus*-associated community was found to be present in both modern and ancient individuals, with functions linked to carbohydrate and dairy metabolism. These functions were missing from the *Methanobrevibacter*-associated community, suggesting that a change in diet, specifically carbohydrate and dairy consumption, after the Industrial Revolution may have impacted the continuity of these oral communities [130]. Due to the abundance of socio-economic changes occurring during a short period of time during the Neolithic and Industrial Revolution and the incomplete evidence of the archaeological record, it is impossible to control for all variables and therefore determine if the cause of a certain change in the oral microbiome was due to the introduction of animal husbandry, new dietary resources, new trade routes or a myriad of other factors (Box S1, available in the online version of this article). The evidence provided by functional profiles in ancient oral microbiomes lends to the specificity of information we can deduce about the relationships between host and microbial community in ancient contexts.

Moving into the twentieth century and beyond, changes in diet, the lasting impact of the Industrial Revolution and the excessive use of antibiotics are all thought to have contributed to the reduction in biodiversity and greater abundance of pathogenic bacteria in the modern oral microbiome [131]. The dramatic increase in sugar consumption since the Industrial Revolution probably contributed to the predominance of oral diseases such as caries and periodontal disease in the twentieth and twenty-first centuries [38,132, 133]. There is currently little information about changes or stability in the human oral microbiome during with twentieth century, as most studies focus on post-industrialization in the nineteenth century or modern, living individuals in the twenty-first century.

### Disease

At a physiological level, dental disease such as caries is routinely examined in the archaeological record. This provides an understanding of the nature of physical manifestations of dental disease. Due to these macroscopic investigations it is known that dental disease was generally less frequent in hunter-gatherers [134,136], increased in many populations that adopted agriculture [119,134, 137] and then further increased in prevalence in populations that have undergone industrialization [38,138]. To gain evidence of dental disease in ancient oral microbiomes it must be possible to observe a change in the characteristics of the bacterial communities between healthy and diseased oral environments via the aDNA preserved in dental calculus. In modern contexts, progression of oral diseases is thought to be associated with a number of causes, including enrichment of red complex pathogens [34,139] and the functional activity of the microbial community [140]. In ancient contexts the connection between changes in the oral microbiome and prevalence of oral disease can be more difficult to identify [141], but several studies have made headway.

aDNA isolated from 5700-year-old chewed birch pitch from southern Denmark was used to infer hunter-gatherer characteristics such as biological sex, geographical heritage, appearance and disease [142] (Fig. 2). The study identified red complex bacteria, suggesting this individual may have had a severe type of periodontal disease. In this instance, when a physical examination of disease was not possible, genetic evidence provided information about the potential health status of this ancient individual. Red complex bacteria suggesting periodontal disease have also been identified in mummified individuals from ancient Egypt [143], in pre-Hispanic and Colonial individuals from Mexico [144], in ancient and modern individuals from Sardinia [145], and in Medieval individuals from Germany [146].

A combined genomic and proteomic analysis of the oral microbiome composition of two Palaeolithic hunter-gatherer individuals from Italy compared to modern humans showed a shared microbial community between ancient calculus and modern plaque [147]. Interestingly, diseased modern samples were characterized by increased Gram-negative, anaerobic bacteria. The ancient samples exhibited similar characteristics, hinting at the possibility of dental disease for those ancient individuals [147]. These findings are contradicted by Velsko *et al*. [141], who found that there were no patterns linking a state of dental disease with changes in the composition or function of the oral microbiomes of smokers and non-smokers in post-Medieval Netherlands.

Additionally, there are several different diseases that manifest throughout the body that have been identified based on genetic preservation in dental calculus. Furthermore, genetic identification of disease can be useful for pathogens that kill the host before there is manifestation of that disease on the bone, which therefore cannot be observed macroscopically in the archaeological record. The ability to detect disease in archaeological remains when it is not visible on the bone allows for a greater depth of understanding of the disease burden across multiple periods throughout history. For example, an ancient *Mycobacterium leprae* genome, the leprosy-causing bacteria, was identified and authenticated from the dental calculus of a sixteenth century Norwegian individual [148]. Physical evidence of leprosy could not be confirmed on the skeletal remains, but this genetic evidence lends favour to identifying the presence of this disease [148].

### The evolution of AMR mechanisms in the oral microbiome

When considering the functional genetic repertoire of oral microbial communities, AMR is an important factor, and exploring their ancient evolution is an important step to understanding which factors may prompt an increase or change in ARGs over time. For example, the discovery and phylogenetic analysis of a novel class B beta-lactamase family displaying high penicillinase activity discovered in tenth and twelfth century medieval dental calculus demonstrates its high historical divergence [149]. Exploring the evolution of particular ARGs like these on the sequence level may help us understand what prompted these changes in oral ARGs throughout time.

To briefly explain ancient AMR, ARGs have been observed in a range of ancient, non-pathogenic environments, such as caves [150,151], arctic soil [152] and permafrost [153,154]. However, given the complex ecological nature of antibiotics in microbial ecosystems, the discovery of ARGs in ancient non-clinical settings is not surprising [155,156] because it is naturally occurring [157]. In order to survive, bacteria have always created defence systems that involve producing chemicals to defend themselves against other micro-organisms, as well as resistance mechanisms to protect themselves from the same microbes [158], and the majority of therapeutically relevant antibiotic classes are generated from natural sources [159], for example the antibiotic-producing (macrolides) and soil-borne bacteria *Streptomyces*, which are thought to be approximately 380 million years old [160]. *Streptomyces* are omnipresent in the natural environment and have been found in soil communities [161,162], hailstone and rainwater [163], and salty atmospheres [164], and in extreme environments mostly void of humans such as the Arctic [165], the Antarctic [166,167] and desert lands [168]. Therefore, it is not suspiring to learn that ARGs encoding macrolide resistance have been identified in a range of ancient habitats such as permafrost, coprolites, mummified individuals [149] and historical dental calculus samples [146,149], as *Streptomyces* have always been around to cause selective pressure. Based on this, it could be argued that a high abundance of macrolide ARGs in ancient oral samples was due to close human proximity to the micro-organisms that create them. Oral bacteria could have been regularly exposed to *Streptomyces* through a hunter-gatherer or agriculturalist herbaceous diet (food derived from plants, such as grains, legumes and cereals), rather than one high in processed foods, refined sugars and artificial additives.

There has always been selective pressure that causes bacteria to confer resistance, and not just by antibiotics, but by other substances such as heavy metals which are known to operate as co-selecting agents in the spread of antibiotic resistance in human pathogens across various environments [169] including modern dental calculus [170]. Bacteria have devised many targeted resistance mechanisms to combat metal(loid)s [171], which are worth exploring across historical periods, such as the Industrial Revolution, when heavy metal pollution affected communities differently depending on their economic affluence and social class [172]. With a focus on zinc, as it is known to co-select for antibiotic resistance, [173] showed that antibiotic-resistant bacteria were enriched in metal-contaminated pond sediments dating to the Industrial Revolution, indicating the pervasive effects of metal–antibiotic co-selection in the environment. Therefore, Industrial Age calculus also has the potential to encapsulate bacterial genes conferring resistance to metals, known as metal resistance genes (MRGs), which might be investigated as initial selection pressures for ARGs. The global effects of historical pollution are not just limited to Industrial Revolutions. Pre-Industrial metal pollution in the atmosphere and in human remains can be traced back to the Roman exploitation of metals [174,178] and prehistoric caves [179,182]. These co-selective processes across time merit investigation to perceive how cultural transitions cause historical changes in AMR. Moreover, it may stimulate discussion over whether pollution should be explored as a contributing factor to the spread of AMR and thus be included in long-term AMR reduction plans.

One study that investigated ARGs across Southern European chronological groups showed that tetracycline resistance was more abundant in modern and historical samples, whereas vancomycin resistance was more abundant in the prehistoric and historical groups [121] (Fig. 3). Questions remain about how these variations of ARGs evolved and whether later versions evolved from earlier versions to adapt to new environments or if they evolved separately [121]. Individuals from medieval Germany also show evidence of ARGs [146] such as aminoglycosides, β-lactams, bacitracin, bacteriocins and macrolides, in addition to genes for multidrug efflux pumps. Interestingly, *Tannerella forsythia* was found to be devoid of resistance genes. This demonstrates that this pathogen had not yet acquired resistance mechanisms in the distant past, as evidenced by a wide gap in its genome reconstruction. On the other hand, a study of individuals from Mexico revealed no ARGs in ancient individuals, while they were identified in modern individuals [144].

These studies raise questions, such as why one finds an abundance of tetracycline resistance genes [121] while the other indicates that tetracycline ARGs had not yet developed [146], both in historical European samples. In this instance, questioning the specificity of historical periods is important in the context of AMR evolution because it may aid in explaining these findings. To elaborate, Ottoni *et al*. [121] identified putative tetracycline ARGs that date back to the eighteenth century, while Warinner *et al*. [146] found a lack of these particular ARGs in earlier medieval samples (*c*. 950–1200 CE). This should encourage research to investigate what happened between these two historical periods in Europe that may have triggered tetracycline resistance in oral bacteria. There are alternative explanations for the discovery of these genes. Tetracycline resistance is a prevalent characteristic in modern oral pathogens, as previously stated; as such, its presence could be the result of modern contamination. The potential misinterpretation of environmental ARGs as a result of improper decontamination protocols has been suspected [183] in a study on ancient mummies [184], which demonstrates how ARGs can be erroneously interpreted in ancient samples.

Contamination is one of many reasons why identifying putative ARGs from ancient remains can be challenging, as well as the fact that aDNA reads are more difficult to recover due to degradation and fragmentation. Furthermore, we only acquire information on the most recent common ancestor; no references are provided for query sequences associated with extinct ancient mechanisms of AMR, as they do not exist. The increase in modern antibiotic usage in the environment and in clinical settings are why we see abundant ARGs in contemporary oral microbiomes in comparison to ancient ones, but the above issues in the identification of ancient ARGs make it more difficult to infer resistance from archaeological remains.

There are other ways to explore ARGs in clean models. For example, one investigation has uncovered ARGs in the oral microbiomes of a secluded, previously uncontacted Yanomami Amerindian community, despite having been isolated for possibly over 11 000 years in South America with no known exposure to antibiotics [73]. This is an example of how researchers can learn about the evolution of antibiotic resistance in human oral microbiomes void of human-made antibiotic influence, and whether ARGs are innate or exogenous traits of the oral cavity. Using dental calculus from other mammals also gives insight into acquired ARGs in the oral cavity prior to the widespread use of modern antibiotics, which can help towards improving strategies to control the spread of ARGs in the environment. For example, one study used dental calculus from wild bears to trace changes in AMR and quantify the diversity of ARGs from before the antibiotic era, stressing that human activity impacts diverse microbial communities in wild animals and the environment [185]. It is noteworthy that mammals are particularly useful when researching the transmission of ARGs in the environment to the clinic; for example, hedgehogs have demonstrated how clinically significant resistance genes exist in the natural world and how human pathogens can acquire them [186].

The analysis of ARGs in ancient oral microbiota is rarely explored and further research should take advantage of ancient dental calculus as a useful source of ARGs. Studying ancient ARGs has potential to help us understand emerging resistance mechanisms in clinical settings and the wider environment and underscores the responsible use of antibiotics to prevent resistance from spreading further. Overall, by studying resistance mechanisms in ancient oral bacteria, we can investigate the extent to which the oral cavity serves as a focal point for ARGs and their spread. This could lead to a greater focus on the oral microbiome as part of the fight against AMR.

## Conclusion

The established understanding of the human oral microbiome is based on knowledge of the behaviour of individual bacterial species, how bacteria interact with other bacteria within the oral cavity and how bacterial communities respond to external influences. This examination has shown how the significant societal and behavioural changes occurring during the pre-Neolithic, Agricultural Revolution, Industrial Revolution and Antibiotic Era probably influenced the evolution of the human oral microbiome. Furthermore, the genomic and functional profiles of these communities may have shifted over the centuries, but there is currently a gap in knowledge that needs to be filled, particularly from the Industrial Revolution, which has been shown to be a period of immense change. Compared to other habitats and microbiomes, there is a deficit in ARGs in databases and study on ARGs in the ancient human oral microbiome. There is a particularly significant deficiency of research regarding ARGs from the Industrial Revolution. Given the current AMR crisis, it would be worthwhile to investigate ancient resistance mechanisms in bacteria. Further research should concentrate on ancient dental calculus as a valuable source of ARGs, as the extent of their abundance sheds light on acquired ARGs in the oral cavity prior to the widespread use of modern antibiotics. WGS provides the high-quality data necessary to study these genes and their functions in ancient oral microbiomes. Overall, understanding the nature, richness and evolution of resistance in the past offers predictive and comparative value for the future.

## supplementary material

10.1099/mgen.0.001251 Uncited Table S1.

## Abbreviations

aDNA: ancient DNA
AMR: antimicrobial resistance
ARG: antibiotic resistance gene
rRNA: ribosomal ribonucleic acid
WGS: whole genome sequencing
