# Supplementary material for: Inferring diet, disease and antibiotic resistance from ancient human oral microbiomes
Source: Microb Genom. 2024 May 13;10(5):001251. doi: 10.1099/mgen.0.001251 (PMC11165619; doi:10.1099/mgen.0.001251)
Supplement: Uncited Table S1. [file mgen-10-01251-s001.pdf]

Supplementary Box 1: Description of preservation bias and issues affecting the study of microbiomes from archaeological materials.

Investigation of archaeological material is accompanied by the issue of preservation bias, meaning that not all materials will survive in the archaeological record (Jackes 2011) and therefore many variables are impossible to control for because we are unable to determine every detail of an ancient individual's life. Consequently, it may be impossible to control for factors such as diet or profession. Ancient humans were likely to have participated in an array of activities that would be considered factors able to affect the oral microbiome and it is therefore difficult to determine the exact cause of any potential detected changes in the oral microbiome.
